# Knowledge, attitudes, and practices among Syrian dentists towards minimal invasive dentistry and chemomechanical caries removal: a cross-sectional study

**DOI:** 10.1038/s41405-025-00343-7

**Published:** 2025-05-27

**Authors:** Dana Alakkad, Mohammed N. Al-Shiekh, Mawia Karkoutly, Mohannad Laflouf

**Affiliations:** Department of Pediatric Dentistry, Faculty of Dentistry, Damascus University, Damascus, Syrian Arab Republic

**Keywords:** Dentistry, Occupational health

## Abstract

**Objectives:**

This study aimed to evaluate and compare the knowledge, perceptions, attitudes, and clinical experiences of general dental practitioners (GPDs), pediatric dentists (PDs), and other dental specialists (ODSs) regarding minimal invasive dentistry (MID) in Damascus, Syria.

**Materials and methods:**

It was an observational, quantitative study utilizing a questionnaire-based online survey. Demographic information data collected included gender, age, specialty, years of practice, number of patients treated per day, and daily working hours. The knowledge assessment evaluated participants’ understanding of MID, their awareness of preventive treatment procedures, previous training, and sources of knowledge. Subsequently, attitude toward MID and chemomechanical caries removal (CMCR) questions assessed participants’ perspectives on MID principles and their stance on CMCR. In addition, clinical practices explored the participants’ application of CMCR in their daily practice. Descriptive statistics were utilized to illustrate the frequency and percentage of categorical variables. A chi-square test was performed to investigate the relationship between specialization, years of experience, and knowledge of the MID and CMCR. *p*-value below 0.05 was deemed statistically significant.

**Results:**

A total of 252 participants were included in the study. According to MID’s knowledge level, most respondents agreed on the importance of fluoride for remineralization (90.1%) and using sealants for high caries-risk children (64.7%). Caries risk assessments (CRA) are strongly agreed (90%). A small percentage of dentists reported rarely using the CRA technique. However, approximately one-third of them consistently evaluated patients’ dietary habits. Additionally, 30.2% frequently assessed the patient’s current fluoride exposure. 67.5% were familiar with the CMCR technique. The chi-square test revealed a significant relationship between knowledge and years of experience, particularly for those with 0–2 years of experience (*p* = 0.006). However, specialization did not impact knowledge, as indicated by the *p* = 0.076. Furthermore, the chi-square test showed that years of experience did not significantly affect knowledge of the technique.

**Conclusions:**

This research provides important perspectives on how dental professionals are adopting MID and CMCR. Although practitioners received MID training during their university studies, only a small percentage expressed complete confidence in their abilities, indicating that dental training programs could be enhanced. It was also identified a strong consensus regarding the advantages of fluoride and preventive measures. However, views varied concerning specific diagnostic and treatment techniques, particularly ART and pit and fissure sealants in permanent teeth.

## Introduction

Recent efforts were directed toward tooth structure preservation through a better understanding of the mechanism of dental caries as an infectious condition [1]. Therefore, Minimal Invasive Dentistry (MID) has emerged as an alternative to preserve and interrupt the disease progression [2]. MID includes caries risk assessment, caries management by risk assessment (CaMBRA), different diagnostic tools, and management of non-cavitated and cavitated lesions [2, 3]. MID interventions include fluoride Casein phosphopeptide amorphous calcium phosphate (CPP-ACP), xylitol, and silver diamine fluoride (SDF) application [2, 4]. In addition, it involves pits and fissure sealants, atraumatic restorative treatment (ART), preventive resin restoration (PRR), tunnel preparation, laser, air abrasion, polymer burs, and chemomechanical caries removal (CMCR) [2, 5]. CMCR represents a promotion in caries removal methods in the 1990s, and chemical agents have been developed in the past 10 years, causing a breakthrough. In addition, it helps alleviate pain during dental treatment, a cornerstone in pediatric dentistry work [6].

CMCR is known as the process of softening decayed dentine chemically and subsequently removing soft carious structures using manual dental instruments in an atraumatic mechanical force without any alterations of both affected dentine and healthy dentine [5, 6]. Thus, it is considered a less destructive approach that reduces the risk of exposing the pulp during cavity preparation by conventional rotary instrumentation [5, 6]. The CMCR technique can be highly beneficial in anxious patients, medically compromised including patients with disability, special health care needs (SCHN) patients, and elderly or pediatric patients [6, 7]. In addition, it can be used in public health sectors, offering many advantages, mainly avoiding patient discomfort in their dental care by minimizing the noise and vibration, pain stimulus, and the need for a local anesthetic [6, 7]. SCHNs who exhibit deviation from typical health standards necessitate attention and particular strategies, and CMCR can be an appropriate choice [6, 7].

Despite the increasing focus on MID, there remains a significant deficiency in the understanding and practical experience with CMCR agents among dental professionals. A survey of dental professionals in Riyadh and AlKharj, Saudi Arabia, revealed that although general dental practitioners (GDPs) know the benefits of MID, there were notable deficiencies in their attitudes towards caries detection methods and the application of minimally invasive procedures. Furthermore, dental companies’ limited availability, marketing, and promotion of CMCR products have further hindered their widespread adoption in developed and developing countries [8]. It highlights an urgent need to evaluate practitioners’ knowledge, attitudes, and experiences regarding CMCR techniques to identify barriers to their implementation. Therefore, this questionnaire aimed to evaluate and compare the knowledge, perceptions, attitudes, and clinical experiences of GDPs, pediatric dentists (PDs), and other dental specialists (ODSs) regarding MID in Damascus, Syria. Such research reveals critical gaps in students’ understanding of MID topics within their academic program, which may be a potential alternative treatment modality in future dental practice. It, in turn, allows educational institutions to recognize these deficiencies and take measures to address them effectively [9]. The null hypothesis suggests that there would be no statistically significant differences observed among the knowledge, perceptions, attitudes, and clinical experiences of GDPs, PDs, and ODSs concerning MID in Damascus, Syria.

## Materials and methods

### Study design

This study utilized an observational, quantitative approach through a questionnaire-based online survey. The Institutional Ethical Committee of Damascus University provided the ethical approval (N578/2025). The study adhered to the principles outlined in the Declaration of Helsinki [10] and to STROBE checklists [11]. The survey was conducted between October and December 2024, ensuring that participation was voluntary and anonymous. Written informed consent was acquired from every participant after providing a thorough explanation of the study’s objectives.

### Sample size and participants

The required sample size was determined using a one-proportion formula [12]:

n_0_ = z^2^⋅p⋅(1−p)/e^2^

n_0_: Sample size.

z: z-score.

p: Estimated proportion of the population.

e: Margin of error.

A total of 252 participants were included in the study. The Syrian Dental Association provided a database of dental practitioners in Damascus. The study encompassed GDPs, PDs, and ODSs without restrictions on years of experience. Participants were required to practice in Damascus at the time of the survey.

### Procedures

The questionnaire was developed based on previously validated studies [13, 14] and underwent pilot testing with ten participants to ensure clarity and ease of completion, with an estimated response time of no more than 10 min. It was designed in Arabic and distributed online via Google Forms, with the survey link shared through social media platforms, including WhatsApp, Facebook, and Instagram. Each participant received a detailed information letter outlining the study’s objectives and rationale before proceeding with the questionnaire.

### Questionnaire structure

The survey consisted of five sections. First, a brief overview of minimally invasive dentistry (MID), along with a statement emphasizing the voluntary and anonymous nature of participation. Demographic information data collected included gender, age, specialty, years of practice, number of patients treated per day, and daily working hours. The knowledge assessment in the third section evaluated participants’ understanding of MID, their awareness of preventive treatment procedures, previous training, and sources of knowledge. Subsequently, Attitudes toward MID and CMCR questions assessed participants’ perspectives on MID principles and their stance on CMCR. In addition, clinical practices explored the participants’ application of CMCR in their daily practice. The questionnaire covered key aspects of caries management, including diagnosis, risk assessment, preventive strategies, remineralization therapy, and ultra-conservative restorative approaches. Several items were structured using a Likert-type scale to gauge participants’ levels of agreement regarding diagnostic, preventive, and restorative techniques. This methodological approach ensured that the study captured comprehensive insights into the knowledge, attitudes, and clinical practices of dental practitioners in Damascus regarding MID and CMCR [13, 14].

### Statistical analysis

The Statistical Package for Social Sciences (SPSS) (IBM Corp., Armonk, NY, USA) was utilized for all statistical analyses. A *p*-value below 0.05 was deemed statistically significant. Descriptive statistics were employed to outline the frequency and percentage of categorical variables. A chi-square test was conducted to explore the association between specialization and years of experience and knowledge of the MID and CMCR.

## Results

A total of 252 participants were included in the study. The majority are female (67.5%). A significant proportion (86.5%) are under 30, with smaller percentages in the older age groups. In addition, over half of the participants (54.4%) have 0–2 years of professional experience, and this number decreases as years of experience increase (Table 1). The majority of participants are general dentists (64.3%), followed by pediatric dentists (18.7%) and those from other specialties (17.1%). Also, more than half of the participants (54%) work 1–4 h per day, with fewer working longer hours (Table 1). Regarding MID training, 62.3% have received some form of training, though only 25% report being thoroughly trained, and 12.7% have not received any training. Most participants received their MID training during university (44.8%), with smaller groups trained during specialization (18.7%) or clinical practice (23.8%). The majority of participants were trained through lectures (76.6%), with significant numbers also receiving information from Professional colleagues (48.4%) and in-clinic training (52.4%) (Table 1).

**Table 1:** Demographic and professional characteristics of participants of minimal invasive dentistry [1] training.

		Frequency	Percentage
Gender	Male	82	32.5
	Female	170	67.5
Age	<30	218	86.5
	30–45	21	8.3
	46–60	8	3.2
	>60	5	2.0
Years of experience	0–2	137	54.4
	2–5	76	30.2
	5–10	17	6.7
	>10	22	8.7
Specialization	General Dentist	162	64.3
	Pediatric Dentist	47	18.7
	Other specializations	43	17.1
Number of work hours	1–4	136	54.0
	5–8	99	39.3
	>8	17	6.7
Number of treated patients	<10	221	87.7
	≥10	31	12.3
Are you trained in doing MID?^a^	Thoroughly	63	25.0
	To a certain extent	157	62.3
	Not at all	32	12.7
If yes, when did you receive training?^b^	During university stage	113	44.8
	During specialization stage	47	18.7
	During clinical practice	60	23.8
How was MID taught to you?^b^	Lectures	193	76.6
	Professional colleagues	122	48.4
	In clinic	132	52.4
	Others	7	2.8

^a^Minimal Invasive Dentistry [1].

^b^Values in these variables considered that 12.5 of the sample population never knew about MID.

According to MID’s knowledge level, most respondents agreed on the importance of fluoride for remineralization (90.1%) and using sealants for high caries-risk children (64.7%). However, Atraumatic Restorative Treatment (ART) for high-risk children has significant neutrality (42.1%) and disagreement (18.3%). Also, the effectiveness of pit and fissure sealants in permanent teeth was disagreed (41.3%) (Table 2). The sandwich technique is supported for permanent teeth but less for primary teeth. Caries risk assessments are strongly agreed (90%). Still, opinions on treatments like enameloplasty, fluoride toothpaste, fluoride rinse, CPP-ACP, and xylitol gum are more divided, with many respondents remaining neutral or disagreeing (Table 2).

**Table 2:** Level of knowledge of MID principles based on a five-point Likert scale (values as percentages).

	Strongly agree	agree	Neutral	Disagree	Strongly disagree
There is a direct relationship between carious lesions and intake of carbohydrates	54.8	41.3	1.2	2.4	0.4
Fluoride is an essential agent in the teeth remineralization process	29.4	60.7	4.4	5.2	0.4
Sealant should be used as a routine procedure for high caries-risk children	64.7	27.4	4.4	2.0	1.6
ART could be used with high caries-risk children^a^	10.3	23.8	42.1	18.3	5.6
Sandwich technique is effective in the treatment of caries in permanent teeth^b^	21.8	51.6	14.3	11.5	0.8
Sandwich technique is effective in the treatment of caries in primary teeth^b^	8.7	30.6	32.9	23.8	4.0
Pit and fissure sealant is effective in treating caries in permanent teeth	10.7	18.3	13.5	41.3	16.3
Preventive resin restoration is effective in the treatment of caries in permanent teeth	13.9	34.1	23.4	22.6	6.0
CRA should be conducted with all patients^c^	56.7	33.3	6.3	3.2	0.4
Tunnel preparation is more effective for proximal carious lesions than class II preparation	7.5	21.8	36.9	31.0	2.8
Enameloplasty is an effective way to prevent dental caries	6.0	39.7	44.4	8.7	1.2
Fluoride toothpaste is effective in treating carious lesions in permanent teeth	9.9	29.4	17.1	32.9	10.7
Fluoride varnish is effective in treating carious lesions in permanent teeth	8.7	29.4	18.3	33.7	9.9
Fluoride rinse is effective in treating carious lesions in permanent teeth	6.7	25.4	19.4	38.9	9.5
CPP-ACP is effective in treating carious lesions in permanent teeth^d^	4.8	19.8	58.3	13.5	3.6
Xylitol gum is effective in treating carious lesions in permanent teeth	6.0	21.8	32.1	32.1	7.9

^a^Atraumatic restorative treatment (ART).

^b^Sandwich technique (glass ionomer + composite).

^c^Caries Risk Assessment (CRA).

^d^Casein PhosphoPepetide – Amorphous Calcium Phosphate (CPP-ACP).

Table 3 illustrates the participants’ attitudes toward applying MID principles and protocols in dental clinics. A small percentage of dentists reported rarely using the CRA technique. However, approximately one-third of them consistently evaluated patients’ dietary habits. Additionally, 30.2% frequently assessed the patient’s current fluoride exposure. Regarding using an explorer for detecting dental caries, 12.7% of respondents reported rarely using it. Similarly, 33.7% of participants indicated that they rarely used magnification during dental procedures (Table 3). Regarding of radiograph usage, over half of dentists incorporate them into their daily practice. However, only a small group (9.5%) reported using FOTI for diagnostic purposes (Table 3).

**Table 3:** Distribution of answers to questions assessing attitude towards the application of MID principles and protocols in the dental clinic.

	Always (100%)	Mostly (75–99%)	Often (50–74%)	Sometimes (10–49%)	Never/rarely (0–9%)
CRA^a^	47.2	27.4	11.1	10.7	3.6
Evaluate the patient’s dietary habits	32.1	26.6	19.8	16.3	5.2
Identify current fluoride exposure	17.5	30.2	22.2	18.7	11.5
Plan restorative materials and techniques based on the patient’s CRA	37.7	25.0	17.9	10.7	8.7
Use of explorer that is not sharp	33.7	25.8	16.3	11.5	12.7
Magnification (loupes/microscope)	11.1	17.1	19.0	19.0	33.7
Radiographs	52.0	31.7	10.7	4.0	1.6
Light transmission (FOTI: Diagnodent)	9.5	14.3	17.9	19.0	39.3

^a^Caries Risk Assessment (CRA).

Table 4 summarizes dental professionals’ awareness, knowledge, and attitudes toward the CMCR technique for removing dental caries. 67.5% were familiar with the technique, mainly learned from university lectures (70.5%) and books (42.9%). The perceived benefits include being conservative (66.4%) and a good choice (61.1%), while disadvantages involve high costs (50%) and limited availability (46.4%). In daily practice, 63.6% never used this technique, and 75.3% believed it was effective. Most respondents rated patient satisfaction as “natural” (53%) or “good” (33.5%). The technique was considered more time-consuming by 47.7% of respondents, and 59.4% felt it reduced the need for local anesthesia (Table 4). Regarding materials, Carisolv (50.5%) was most preferred. In comparison with traditional methods, 42.3% think it was not superior, and 60.6% preferred other minimally invasive techniques, like Laser and ART (Table 4).

**Table 4:** Distribution of answers related to awareness (knowledge, practice, and attitude) about mechanical-chemical technique for removing dental caries.

		Frequency	Percentage
Are you familiar with mechanical-chemical technique for removing dental caries?	Yes	170	67.5
	No	82	32.5
Sources of that knowledge:	Books	73	42.9
	Journals	23	13.5
	Webinars	21	12.3
	University lectures	120	70.5
	Conferences	46	27.0
	Training courses	16	9.4
	Others	7	4.1
What are the advantages of this technique?	Easy to use	53	31.1
	Patient’s cooperation	49	28.8
	Good choice	104	61.1
	Conservative technique of removing dental caries	113	66.4
	Time-saving	13	7.6
	None of the above	10	5.8
What are the disadvantages of this technique?	Expensive	85	50.0
	Not available	79	46.4
	Ineffective	10	5.8
	There is little information on this topic	44	25.8
	Time-consuming	59	34.7
	Maybe I’ll try it a little	32	18.8
	None of the above	6	3.5
Do you use this technique in daily practice in your clinic?	Never	108	63.6
	Rarely	29	17.0
	Sometimes	25	14.7
	Based on the patient’s choice	8	4.7
Can mechanical-chemical removal of dental caries be used in children and adults?	Yes	115	67.6
	No	13	7.6
	I don’t know	42	24.8
Do you think that the mechanical-chemical technique of removing dental caries is effective?	Yes	128	75.3
	No	42	24.7
How do you rate patient satisfaction with this technique?	Bad	4	2.3
	Natural	90	53.0
	Good	57	33.5
	Very good	19	11.2
Depending on the time, do you think this technique:	More time-consuming	81	47.7
	Less time-consuming	17	10.0
	Medium time-consuming	41	24.1
	I’m not sure	28	16.5
	None of the above	3	1.7
Does this technique reduce the need for local anesthesia during caries removal?	Yes	101	59.4
	No	38	22.3
	I don’t know	31	18.3
Have you experienced any unwanted effects when using this technique on children?	Yes	22	12.9
	No	148	87.1
Do you think that the mechanical-chemical technique of removing dental caries is better than the traditional technique?	Yes	52	30.6
	No	72	42.3
	I don’t know	46	27.1
Which materials do you prefer for the mechanical-chemical removal of dental caries?	Brix3000	22	13.0
	Caridex	36	21.1
	Caricare	5	2.9
	Carisolv	86	50.5
	Papacarie	5	2.9
	Sodium hypochlorite	25	14.7
	None of the above	35	20.6
Instead of mechanical-chemical removal of dental caries, do you prefer other MID techniques?^a^	Yes	103	60.6
	No	22	12.9
	I don’t know	45	26.5
What are these techniques?	Air abrasion	32	31.1
	Laser	44	42.7
	ART^b^	43	41.7
	SDF^c^	23	22.3
	Smart bur	31	30.1
	None of the above	5	4.8

^a^Minimal Invasive Dentistry [1].

^b^Atraumatic Restorative Treatment (ART).

^c^Silver Diamine Fluoride (SDF).

The chi-square test revealed a significant relationship between knowledge and years of experience, particularly for those with 0–2 years of experience (*p* = 0.006). However, specialization did not have a statistically significant impact on knowledge, as indicated by the *p*-value = 0.076 (Table 5).

**Table 5:** Chi-square test to investigate the relationship between (specialization and Years of experience) and knowledge of the MID.

		Knowledge of the MID						*p*-value
		Thoroughly		To a certain extent		Not at all		
Years of experience	0–2	24	17.5	89	65.0	24	17.5	.006^*^
	2–5	21	27.6	49	64.5	6	7.9	
	5–10	9	52.9	7	41.2	1	5.9	
	>10	9	40.9	12	54.5	1	4.5	
Specialization	General Dentist	32	19.8	105	64.8	25	15.4	.076
	Pediatric Dentist	15	31.9	28	59.6	4	8.5	
	Other specializations	16	37.2	24	55.8	3	7.0	

^*^a significant difference.

Furthermore, the chi-square test showed that years of experience did not significantly affect knowledge of the technique, as the *p*-values for all experience groups were above 0.05. However, specialization had a significant relationship, with PDs showing the highest level of knowledge (87.2%), while GPDs and those in ODSs had lower knowledge levels (62.3% and 65.1%, respectively) (Table 6).

**Table 6:** Chi-square test to investigate the relationship between (specialization and Years of experience) and knowledge of CMCR.

		knowledge of CMCR					*p*-value
		Yes			No		
Years of experience	0–2	85	62.0	52		38.0	.150
	2–5	58	76.3	18		23.7	
	5–10	13	76.5	4		23.5	
	>10	14	63.6	8		36.4	
Specialization	General Dentist	101	62.3	61		37.7	0.005^*^
	Pediatric Dentist	41	87.2	6		12.8	
	Other specializations	28	65.1	15		34.9	

^*^a significant difference.

## Discussion

This study offers valuable insights into the current adoption of MID and CMCR among dental professionals. A significant agreement was found regarding the benefits of fluoride and preventive strategies. Nonetheless, opinions differed on particular diagnostic and treatment methods, especially ART and applying pit and fissure sealants on permanent teeth. While many practitioners underwent MID training in their university programs, only a limited number reported full confidence in their skills. CMCR and MID are globally recognized for their conservative approaches to managing dental caries [2, 6]. However, it remains uncertain whether these methods will be embraced in low-income nations such as Syria. This study explored the awareness, attitudes, and application of these techniques among dentists in Syria. Thus, an online survey was adapted from previous questionnaires to ensure validity and reliability. The findings of this research can contribute to improving dental education and training programs by pinpointing obstacles to implementation and areas where knowledge is lacking, ultimately facilitating the integration of MID methods into standard dental practice. Given that PDs are a crucial component of dental care for children, they were included in this research. Their experiences, feelings about pain, and treatment outcomes are vital in assessing the effectiveness and acceptance of various methods for caries removal [6, 7]. In addition, understanding their role in advancing less invasive treatment approaches can help reduce anxiety and discomfort [6, 7]. GDPs and ODSs were incorporated to gain a comprehensive understanding of the knowledge, attitudes, and clinical practices in this area. Since GDPs often act as the first point of contact for patients, their methods for managing caries hold significant importance. PDs are specialists in children’s oral health and provide valuable insights into treatment strategies that cater to each child’s requirements [6, 7].

The findings of this study provide valuable insights into the knowledge, attitudes, and practices regarding MID and CMCR concepts among Syrian dentists. The sample population was predominantly young, with 86.5% of respondents aged under 30 years. This demographic likely reflects the distribution of younger dentists in academic and professional circles, such as universities and dental societies. Additionally, 54.4% of participants had 0–2 years of experience, indicating that many were recent graduates with current theoretical knowledge. In contrast, only 8.7% had more than ten years of experience, suggesting that MID may not be widely adopted by more experienced professionals, who may be more familiar with traditional methods like G.V. Black’s extension-for-retention concept. The study’s sample was predominantly composed of GPDs (64.3%), which mirrors the general makeup of the Syrian dental community. Most participants (54.0%) reported working 1–4 h daily, with 39.3% working 5–8 h. Only 6.7% worked over 8 h a day, which suggests a diverse range of work environments and schedules. This variation may influence how dentists integrate new concepts like MID into their daily practices and patient care routines.

Regarding training, 62.3% of participants reported receiving some form of training in MID, with 25.0% stating they were thoroughly trained. However, 12.7% had no training in MID, indicating that there is still room for improvement in incorporating MID into dental education. Notably, 44.8% received their training during their university studies, suggesting that MID principles are beginning to be integrated into dental curricula, a positive development for future dentists. The research additionally investigated participants’ understanding of important concepts related to caries. A significant portion (54.8%) of those surveyed acknowledged the connection between tooth decay and carbohydrate consumption, aligning with results from previous studies. The role of carbohydrates, particularly sugars like sucrose, in the development of dental caries is well-established in cardiology. Research supports that high sugar intake contributes to caries, as sugars are fermented by bacteria in the mouth, producing acids that demineralize tooth enamel, leading to early childhood caries (ECC) [15, 16].

Regarding preventive measures, 92.1% of participants agreed on the importance of fluoride in remineralizing teeth. However, only 39.3% recognized the benefit of fluoride varnish in permanent dentition despite evidence supporting its efficacy in both primary and permanent teeth. Studies show that fluoride varnish can prevent dental caries by up to 70%, with the highest effectiveness observed in young children and for occlusal caries in primary teeth and first permanent molars [17–19]. In addition, 24.6% of participants agreed that CPP-ACP is effective in remineralization and enamel defect repair. This relatively low recognition may be attributed to limited exposure to CPP-ACP products or insufficient education on its benefits. However, studies have shown that CPP-ACP can inhibit enamel demineralization by up to 50%, offering remineralization comparable to fluoride treatments [20–22]. Moreover, only 27.8% of respondents were aware of the effectiveness of xylitol gum in preventing caries in permanent dentition. This low percentage may reflect a lack of understanding of xylitol’s role, particularly in permanent teeth. Based on participants’ attitudes towards MID principles and protocols in the dental clinic, the majority of dentists assess caries risk for more than 50% of their patients. This assessment is recognized as one of the five key components of MID strategies [6]. Furthermore, it serves as a crucial element in developing personalized treatment plans for each patient, aiding the patient and dentist in managing and preventing dental caries, particularly for individuals with a high risk of caries development [6].

In the area of patient dietary habits, 57.7% of participants reported using this tool frequently in their practice. However, a significant percentage did not utilize it a lot, indicating a need to improve awareness and adoption of this approach. Evaluating nutritional intake is a critical method for predicting the incidence of dental caries, as high sugar consumption increases the likelihood of caries formation [15, 16]. According to Joury et al. [23] study, the Syrian crisis has led to an increase in sugar consumption among children, highlighting the urgent need for such tools to monitor and control sugar intake in this country. Over half of the dentists in this survey reported using the dental explorer frequently, despite its limitations in visual assessment of dental caries. While the dental explorer has traditionally been employed to detect carious lesions, its effectiveness is debated. It may not reliably indicate caries, as it could simply fit into a fissure. It can cause damage to fragile, demineralized surfaces and potentially spread cariogenic bacteria deeper, reaching areas beyond regular oral hygiene [24–26]. The explorer may also transfer bacteria between infected and healthy. With low sensitivity, it is often an unreliable diagnostic tool [24–26]. As a result, radiographic examinations are typically required to confirm any findings in the clinical examination. Notably, a small percentage (1.6%) of dentists reported never using this method.

CMCR techniques are widely regarded as one of the most common minimally invasive dentistry methods, involving the gentle removal of softened dentin using hand instruments [6]. However, a significant proportion of dentists (32.5%) in this study reported being unfamiliar with these techniques. More than two-thirds of participants believed CMCR is a conservative approach for treating caries and preserving tooth structure. Numerous studies support this view, emphasizing the technique’s goal of minimizing tooth damage [5–7]. CMCR offers an effective alternative to traditional surgical methods, which often involve excessive tooth preparation, risking pulp exposure, pain, and heat generation that can lead to pulp damage [5–7]. However, nearly half of the participants in the study reported that they found this technique expensive. Despite this, many agents used are inexpensive, such as sodium hypochlorite (NaOCl) gel at concentrations of 2.25-2.4% [5, 27]. About one-third of participants reported that they had never used or were unaware of the application of CMCR in children. Overall, CMCR is more commonly used in adults than in children. However, this technique offers a comfortable and painless method for caries removal, making it an excellent option for caries excavation in anxious children and patients with special needs [5–7, 25].

Regarding time consumption, 47.7% of dentists confirmed that CMCR takes more time compared to traditional caries removal. Several studies have also noted that this method is more time-consuming. In general, CMCR is painless and does not require local anesthesia [5–7, 25]. While 59.4% of participants agreed with this, a notable percentage expressed the opposite view. According to the study by Maashi et al. [6], two types of materials can be used as CMCR agents: NaOCl-based agents, such as GK-101e (Caridex) and Carisolv, and enzyme-based agents, such as Papacarie, Biosolv, and Brix3000. In this study, 50.5% of participants preferred Carisolv, which is considered the latest iteration of NaOCl-based CMCR agents and has been sufficiently studied for use in dental practice. However, Carisolv has several drawbacks, including its high cost, unpleasant taste and odor, and the need for specialized curettes [5–7, 25].

While this study provides important insights, there are certain limitations that may have influenced the findings. One potential limitation of these surveys is that dentists may lean towards selecting responses they believe are ‘correct’ instead of those that truly reflect their opinions. The voluntary and anonymous format of the survey is intended to mitigate this possible influence. Although efforts were made to ensure a representative sample, the predominance of younger dentists suggests that the perspectives of more experienced practitioners may not have been fully captured. Additionally, while the questionnaire was designed based on validated studies and pilot-tested, it primarily captures theoretical knowledge rather than direct clinical application, which could be better assessed through observational studies. It is also worth considering that factors such as financial constraints, patient preferences, and institutional policies may play a role in the adoption of MID principles, yet these aspects were beyond the scope of this study. More research is needed to gain a deeper understanding of how MID practices are being integrated into everyday dental care.

## Conclusions

In conclusion, this study offers valuable insights into how dental practitioners are currently embracing MID and CMCR. Among the 252 dentists who took part, most were young, predominantly female general dentists with relatively few years of experience. This youthful profile may affect their exposure and comfort with newer treatment methods. Even though many received MID training during their university education, only a few felt fully confident in their skills, which suggests there’s room for improvement in dental training programs. It also found a strong majority opinion on the benefits of fluoride and preventive strategies. Yet opinions diverged regarding certain diagnostic and treatment methods, specifically ART and pit and fissure sealants in permanent teeth.

## Data Availability

The datasets generated during and/or analyzed during the current study are available from the corresponding author on reasonable request.
